# Correlation between superfluid density and transition temperature in infinite-layer nickelate superconductor Nd_1–*x*_Sr_*x*_NiO_2_

**DOI:** 10.1093/nsr/nwag068

**Published:** 2026-01-31

**Authors:** Zhijie Li, Ruozhou Zhang, Minghui Xu, Keyi Liang, Yan Zhao, Qiushi He, Qizhi Zhou, Boren Chen, Puhua Zhang, Kaizhong Yao, Hongxu Yao, Liang Qiao, Yihua Wang

**Affiliations:** State Key Laboratory of Surface Physics and Department of Physics, Fudan University, Shanghai 200433, China; State Key Laboratory of Surface Physics and Department of Physics, Fudan University, Shanghai 200433, China; School of Physics, University of Electronic Science and Technology of China, Chengdu 610054, China; State Key Laboratory of Surface Physics and Department of Physics, Fudan University, Shanghai 200433, China; School of Physics, University of Electronic Science and Technology of China, Chengdu 610054, China; State Key Laboratory of Surface Physics and Department of Physics, Fudan University, Shanghai 200433, China; State Key Laboratory of Surface Physics and Department of Physics, Fudan University, Shanghai 200433, China; State Key Laboratory of Surface Physics and Department of Physics, Fudan University, Shanghai 200433, China; State Key Laboratory of Surface Physics and Department of Physics, Fudan University, Shanghai 200433, China; State Key Laboratory of Surface Physics and Department of Physics, Fudan University, Shanghai 200433, China; State Key Laboratory of Surface Physics and Department of Physics, Fudan University, Shanghai 200433, China; School of Physics, University of Electronic Science and Technology of China, Chengdu 610054, China; State Key Laboratory of Surface Physics and Department of Physics, Fudan University, Shanghai 200433, China; Shanghai Research Center for Quantum Sciences, Shanghai 201315, China

**Keywords:** high-temperature superconductivity, infinite-layer nickelates, scanning SQUID microscopy, superfluid density

## Abstract

The strong correlation between zero-temperature superfluid density (${{{\rho }}}_{{{\rm s}}0}$) and transition temperature (${{{T}}}_{{\rm C}}$) is considered a hallmark of unconventional superconductivity. However, their relationship has yet to be unveiled in nickelates due to sample inhomogeneity. Here, we perform local susceptometry on an infinite-layer nickelate superconductor Nd_0.8_Sr_0.2_NiO_2_. The sample shows inhomogeneous ${{\mathrm{\rho }}}_{{{\rm s}}0}$ and ${{{T}}}_{{\rm C}}$ at the micron scale. The spatial statistics for different scan areas reveal a linear dependence of local ${{{T}}}_{{\rm C}}$ on ${{\mathrm{\rho }}}_{{{\rm s}}0}$ for ${{{T}}}_{{\rm C}} > 8\ {{\rm K}}$ and a sublinear dependence for ${{{T}}}_{{\rm C}} < 8\ {{\rm K}}$. Remarkably, the overall relationship is reminiscent of that reported in overdoped cuprate superconductors, hinting at a close connection between them.

## Teaser

This study utilizes scanning SQUID susceptometry to find local correlation between superconducting transition temperature and superfluid density in infinite-layer nickelates similar to that in overdoped cuprates, hinting at a close connection between the two systems.

## INTRODUCTION

In the quest to understand the microscopic origin of unconventional superconductivity, a concerted effort has been made to explore the limiting factor of superconducting transition temperature (${T}_{\mathrm{C}}$) [1]. In contrast to the Bardeen–Cooper–Schrieffer (BCS) superconductors in which ${T}_{\mathrm{C}}$ is determined by the pairing strength [2], the ${T}_{\mathrm{C}}$ of unconventional superconductors is always correlated with the zero-temperature superfluid density (${\rho }_{{\mathrm{s}}0} \equiv {\lambda }^{ - 2} \propto \frac{{{n}_{\mathrm{s}}}}{{{m}^*}}$, where $\lambda $ denotes the London penetration depth, ${n}_{\mathrm{s}}$ denotes the superconducting carrier density and ${m}^*$ is the effective electron mass). The latter encodes the stiffness of the quantum-mechanical phase of Cooper pairs, reflecting the resilience of the superconductor to thermal or quantum phase fluctuations [3–6]. In hole-doped cuprate superconductors, for instance, the Uemura law, i.e. ${T}_{\mathrm{C}} \propto {\rho }_{{\mathrm{s}}0}$, holds in the moderately underdoped regime, which was attributed to strong thermal phase fluctuations [3,7]. As the critical doping at which ${T}_{\mathrm{C}} = 0\ {\mathrm{K}}$ is approached, a crossover from ${T}_{\mathrm{C}} \propto {\rho }_{{\mathrm{s}}0}$ to ${T}_{\mathrm{C}} \propto \rho _{{\mathrm{s}}0}^\alpha $ [$\alpha = z/( {z + D - 2} )$ with *z* the quantum dynamic exponent and *D* the spatial dimensionality] was observed [8–10], providing the evidence for quantum phase fluctuations in strongly underdoped cuprates [11,12]. In the overdoped regime, previous superfluid density measurements of overdoped La_2__–_*_x_*Sr*_x_*CuO_4_ (LSCO) films demonstrated that the relation between ${T}_{\mathrm{C}}$ and ${\rho }_{{\mathrm{s}}0}$ is generally linear with an offset as the doping varies, i.e. ${T}_{\mathrm{C}} = {T}_0 + \alpha {\rho }_{{\mathrm{s}}0}$ (${T}_0$, $\alpha > 0$ being constant parameters), but converts into a parabolic scaling, i.e. ${T}_{\mathrm{C}} \propto \rho _{{\mathrm{s}}0}^{1/2}$, for ${T}_{\mathrm{C}}\ < \ 12\ {\mathrm{K}}$ [13]. The strong correlations between ${T}_{\mathrm{C}}$ and ${\rho }_{{\mathrm{s}}0}$ have also been found in heavy fermion [14], transition-metal dichalcogenide [15] and iron-based superconductors [16–19].

It is natural to pose the same question for the recently discovered high-*T*_C_ superconductor infinite-layer (IL) nickelates [20–22]. After all, the parent compound of IL nickelates shares the same nominal transition-metal 3*d*^9^ electron configuration and crystal structure as the cuprates. But the answer is not obvious due to some nontrivial differences in the nickelates: the absence of long-range magnetic order in the parent compound [20,23–25], the multiband electronic structure [26,27] and the possible mixture of *s*- and *d*-wave pairing [28]. Whether these differences lead to a different nature in the unconventional superconductivity of nickelates remains to be investigated. To this end, it is important to examine quantitatively the correlation between ${T}_{\mathrm{C}}$ and ${\rho }_{{\mathrm{s}}0}$ in nickelates [29] and compare it with that found earlier in cuprates.

The current complexities involved in the material realization of superconducting nickelates make it technically challenging to determine this key relationship. Superconducting IL nickelates *R*_1__–_*_x_*Sr*_x_*NiO_2_ (*R* = La, Pr, Nd) have so far been synthesized via CaH_2_-assisted soft-chemistry topotactic reduction of the perovskite *R*_1__–_*_x_*Sr*_x_*NiO_3_ [20–22]. Such a reduction step is critical for inducing superconductivity in the IL nickelates. However, it also introduces significant lattice distortion and inhomogeneous chemical doping [30–35], which then leads to spatial variation in superconductivity at the micron scale [36]. The inhomogeneity impedes accurate determination of ${T}_{\mathrm{C}}$ and the superfluid density by using conventional bulk measurement techniques such as muon spin rotation or two-coil mutual inductance [7,13]. Hence, a highly sensitive local magnetic probe capable of imaging both ${T}_{\mathrm{C}}$ and the superfluid density is essential.

Here, we employ scanning superconducting quantum interference device (sSQUID) microscopy in magnetometry and susceptibility modes to systematically investigate the spatial distribution of ${T}_{\mathrm{C}}$ and the superfluid density in Nd_0.8_Sr_0.2_NiO_2_ (NSNO) films grown on (001)-oriented single-crystal SrTiO_3_ (STO). Our samples exhibit the relatively high bulk ${T}_{\mathrm{C}}$ of 8.6 K for NSNO on STO reported so far [20,24,37,38]. Yet, we still find the spatial inhomogeneity of ${T}_{\rm C}$ and superconducting diamagnetism in different regions of the film. Noticeably, there are ring-like patterns showing weaker diamagnetism, which have also been observed previously on NSNO films by different groups [36], proving to be generic features of NSNO/STO. By analysing the mappings of ${T}_{\mathrm{C}}$ and susceptibility from different regions on the sample, we further find that the inhomogeneous ${T}_{\mathrm{C}}$ is spatially correlated with local ${\rho }_{{\mathrm{s}}0}$: ${T}_{\mathrm{C}}$ varies linearly with ${\rho }_{{\mathrm{s}}0}$ for regions with ${T}_{\mathrm{C}} > 8\ {\mathrm{K}}$, but exhibits a sublinear dependence on ${\rho }_{{\mathrm{s}}0}$ approximate to ${T}_{\rm C} \propto \rho _{{\mathrm{s}}0}^{1/2}$ for ${T}_{\mathrm{C}} < 8\ {\mathrm{K}}$. These results are reminiscent of those reported in overdoped LSCO films, strengthening the link between the superconductivity of IL nickelates and that of cuprates.

## RESULTS AND DISCUSSION

**Figure 1.**
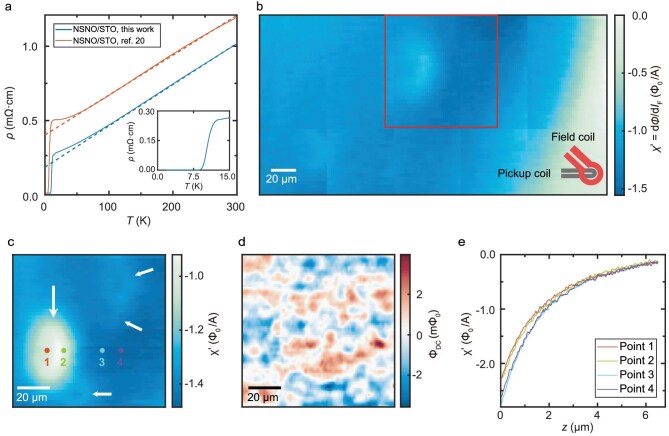
Characterization of superconducting properties of the NSNO film. All data are acquired at ${T}_{{\mathrm{base}}} = 5.00\ {\mathrm{K}}$ unless otherwise specified. (a) Temperature-dependent resistivities of NSNO films. (b) In-phase susceptibility imaging of a region of 280 × 150 μm^2^. Negative $\chi ^{\prime}$ (blue color) indicates diamagnetism. Multiple weakly diamagnetic rings (WDRs) are visible. The square depicts the scan area in panel (c) and Fig. 2. The inset shows the configuration and orientation of the pickup loop and the field coil of the SQUID sensor. (c) In-phase susceptibility imaging of the main WDR (indicated by the large arrow) in panel (b). Smaller WDRs (indicated by small arrows) are visible. (d) DC magnetic-flux imaging of the same region as in (c). (e) Susceptibility-approach curves $\chi ^{\prime}( z )$ of the selected points marked in panel (c).

Two NSNO epitaxial films with thicknesses of 15 and 10 nm were grown on STO (001) substrates by using the pulsed laser deposition technique. No capping layer was introduced for both samples. Details of the sample growth and characterization are depicted in Supplementary Materials S1. The overall high-quality (00*l*)-oriented growth of the films was verified by using atomic force microscopy, X-ray diffraction measurements and scanning transmission electron microscopy (STEM) (Figs S1 and S2). Figure 1a shows the temperature-dependent resistance of one film (the blue line) alongside the data from previous studies (the brown line) [20,39]. The onset temperature of the superconducting transition is ${T}_{{\mathrm{C}},{\mathrm{onset}}}\ = \ 11.5\ {\mathrm{K}}$ and the bulk zero resistance appears at ${T}_{\mathrm{C}} = 8.6\ {\mathrm{K}}$. The linear-in-temperature resistivity is observed in a wide temperature range of ∼100–300 K, which is consistent with previous reports on optimally doped nickelates [39,40]. It should be noted that the ${T}_{\mathrm{C}}$ value and the normal-state transport rely on the type of substrate: NSNO grown on (LaAlO_3_)_0.3_(Sr_2_TaAlO_6_)_0.7_ exhibits higher ${T}_{\mathrm{C}}$ and better conformity of the linear resistivity at low temperatures [39]. Nevertheless, for films grown on the STO substrates, both the ${T}_{\mathrm{C}}$ and the ${T}_{{\mathrm{C}},{\mathrm{ onset}}}$ of this sample are higher and $\frac{{\Delta {T}_{\mathrm{C}}}}{{{T}_{\mathrm{C}}}} = \frac{{{T}_{{\mathrm{C}},{\mathrm{ onset}}} - {T}_{\mathrm{C}}}}{{{T}_{\mathrm{C}}}}$ is smaller than those reported in Refs [20,24,37,38]. Another NSNO film exhibits the highest ${T}_{\mathrm{C}}$ of 12.6 K (Fig. S10) among the NSNO/STO films reported so far (see e.g. [28,41]). These facts indicate the high quality of our NSNO samples.

To characterize the spatial distribution of the superconductivity of the NSNO film, we employ sSQUID magnetometry and susceptometry [19,42–50], which has a high magnetic-flux sensitivity even under a zero magnetic field (see details in Supplementary Materials S2). Figure 1b shows a large-scale (280 × 150 μm^2^) susceptometry mapping measured at *T*_base_ = 5.00 K, which is stitched together from 10 contiguous scans, each with an area of 90 × 90 μm^2^. The film exhibits an overall diamagnetic background, but contains significant inhomogeneity, consistently with a recent SQUID study of IL nickelates [36]. The diamagnetism is weaker on the right edge of the image. There are also two weakly diamagnetic rings (WDRs) with sizes of approximately 30 × 50 μm^2^ (middle) and 20 × 30 μm^2^ (lower-left). From a zoom-in scan of the largest weakly diamagnetic ring, i.e. the main WDR (indicated by the large arrow), multiple smaller WDRs (indicated by small arrows) can be further seen (Fig. 1c): one close to the main WDR and two small rings located at the top-right corner of the scan area. As the susceptibility $\chi ^{\prime}$ is directly proportional to the magnitude of the superfluid density in a thin film [51], these images indicate spatial variation in the superfluid density in the NSNO sample. The inhomogeneous superfluid density can also be found in the second NSNO film with a higher ${T}_{\mathrm{C}}$ (Fig. S10).

We note that, for the susceptometry scan, submicron defects could appear as weakly diamagnetic halos due to the geometry of the nano-SQUID sensor layout [52]. Another possible origin is the formation of a mixed Ruddlesden–Popper secondary phase during the growth process [53], as observed in the STEM cross-sectional images of our sample (Fig. S2). Additionally, loss of oxygen [54] and the presence of hydrogen [35] during the reduction process also affect superconductivity. Considering that the ${T}_{\mathrm{C}}$ of our films is among the highest for NSNO films grown on STO, the observed inhomogeneity may reflect the inherent limitation of the current synthetic method of IL nickelates.

A DC magnetic-flux image shows weak magnetic-domain-like patterns (Fig. 1d) [55]. This is different from the strong flux contrast of isolated superconducting vortices [56]. Such patterns do not show obvious spatial correlation with the susceptometry image (Fig. 1c), suggesting that their origin is different from that of the WDR. Actually, the magnetic-domain-like patterns of the NSNO have also been reported in [36] and were attributed to NiO*_x_* ferromagnetic nanoparticles in that work. Similar magnetic domains with irregular shapes and typical sizes of 5–20 μm were observed from the ferromagnetic EuO layer of a EuO/KTaO_3_ heterostructure, which coexists with the diamagnetism of the interfacial superconductivity [57]. However, the superfluid density in the NSNO film is relatively large so that the overall susceptibility is still diamagnetic. The susceptibility-approach curves $\chi ^{\prime}( z )$ (with *z* representing the distance between the nano-SQUID tip and the sample) on selected points around the main WDR (Fig. 1e) show that the main WDR is also diamagnetic overall. Notably, the value of $\chi ^{\prime}( z )$ on points inside the main WDR (Points 1 and 2) decrease more slowly than those outside the main WDR (Points 3 and 4) as *z* decreases. ${T}_{\mathrm{C}}$ is positively correlated with the diamagnetic strength for these four points, which will be demonstrated in the following.

**Figure 2.**
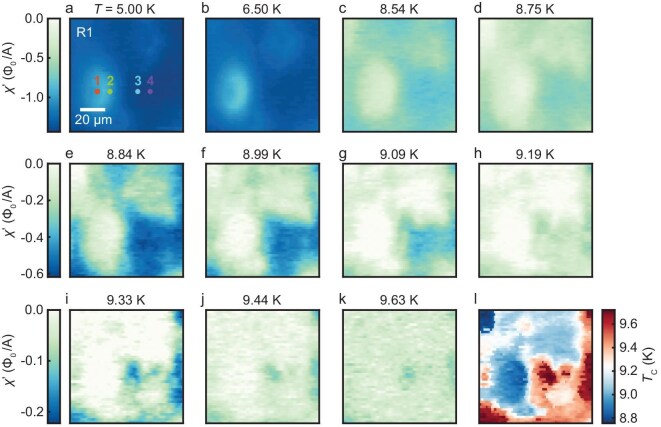
Temperature evolution of the superconducting diamagnetism and map of the local superconducting transition temperature. The scan area, labeled as R1, is the same as in Fig. 1c. (a–k) Susceptometry maps of R1 at different temperatures. All panels in the same row share the same color scale. At *T* < bulk *T*_C_ = 8.60 K, the scan area exhibits diamagnetism with significant inhomogeneity (a–c). At bulk *T*_C_ < *T* ≤ 9.63 K, WDRs and adjacent regions lose diamagnetism quickly with increasing temperature (d–k). Beyond 9.63 K, the diamagnetism of the full scan area vanishes (not shown in the figure). (l) Map of local *T*_C_, which is determined as the onset temperature of superconducting diamagnetism.

The temperature evolution of the susceptometry images over the same scan area of Fig. 1c are shown in Fig. 2. The diamagnetism fades out quickly as the temperature rises from $T = 5.00$ to $8.75\ {\mathrm{K}}$ above the bulk ${T}_{\mathrm{C}}$ (Fig. 2a–d). The average value of $\chi ^{\prime}$ at $T = 8.75\ {\mathrm{K}}$ is only half of that at $T = 5.00\ {\mathrm{K}}$. Moreover, the diamagnetism of the main WDR and upper corners vanishes faster than that of the lower-right region. In the temperature regime of $T\ = \ 8.84$–$9.63\ {\mathrm{K}}$, which is around the bulk ${T}_{\mathrm{C}}$, the non-uniform susceptibility evolution is more obvious. As the temperature increases, the main WDR region becomes non-superconducting ($\chi ^{\prime}\ \sim 0$) first, while the superconductivity in the lower-right region is more persistent, leading to a diamagnetic patch up to $T = 9.09\ {\mathrm{K}}$ (Fig. 2e–g). A few segregated islands remain diamagnetic above $T = 9.33\ {\mathrm{K}}$ (see e.g. the spots near the center of Fig. 2i–k). Superconducting diamagnetic patterns disappear completely for $T > 9.63\ {\mathrm{K}}$ (not shown in the figure). Figure 2l shows the local ${T}_{\mathrm{C}}$ map of the scan area, which is determined as the onset temperature of superconducting diamagnetism ($\chi ^{\prime} < 0$). The local ${T}_{\mathrm{C}}$ is spatially inhomogeneous and the lower values appear mainly inside the WDRs. Again, as no correlation is found between the magnetic contrast (Fig. S4) and the diamagnetic patterns in the susceptibility images (Fig. 2a–k), ferromagnetic clusters are not affecting the local diamagnetism in our samples [34].

To quantify the temperature evolution of inhomogeneity, we calculate the autocorrelation plots of the susceptibility maps at different temperatures (Fig. S5). The typical size of the superconducting patches with constant diamagnetic susceptibilities, ${l}_{\mathrm{p}}$, can be evaluated as the full-width-at-half-maximum of the central peak of the radial profile of the autocorrelation plots (see details in Supplementary Material S4). A similar procedure has been adopted to analysis the gap maps of granular superconductors [58]. As the temperature is increased across the bulk ${T}_{\mathrm{C}}$ of 8.6 K, ${l}_{\mathrm{p}}$ decreases from a nearly *T*-independent value (∼13 μm) to a small but finite value (∼1 μm) (Fig. S6a). The finite value of ${l}_{\mathrm{p}}$ above the bulk ${T}_{\mathrm{C}}$ is in sharp contrast to the disappearance of ${l}_{\mathrm{p}}$ at the bulk ${T}_{\mathrm{C}}$ for a homogeneous superconductor. The former is due to the segregated superconducting patches at high temperatures, corroborating the inhomogeneous ${T}_{\mathrm{C}}$ in the NSNO film. Meanwhile, we find that the temperature dependence of para-resistance above the bulk ${T}_{\mathrm{C}}$ can be well captured by the formula of zero-dimensional Aslamasov–Larkin fluctuations [59], which again indicates the existence of small-sized superconducting patches at high temperatures (Fig. S6b). All these analyses are consistent with the observed inhomogeneous ${T}_{\mathrm{C}}$ in the NSNO film (Fig. 2l).

**Figure 3.**
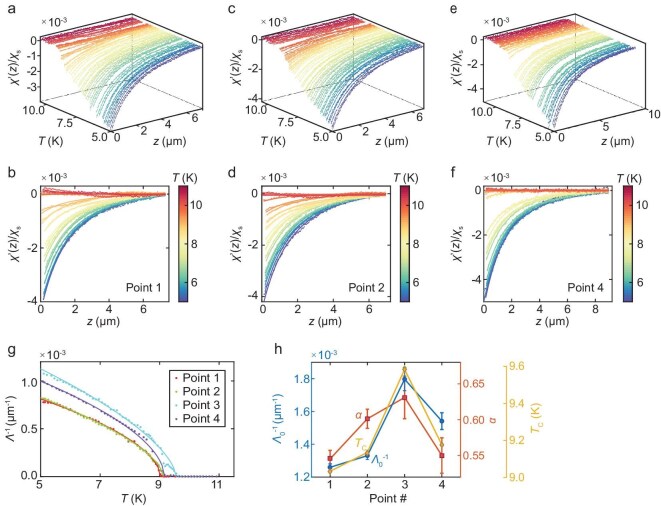
Temperature evolution of susceptibility-approach curves and superfluid density at different points. (a–f) Susceptibility-approach data measured at the points labeled in Fig. 1c: (a, b), (c, d) and (e, f) correspond to Point 1, Point 2 and Point 4, respectively. The colors of the data points represent the temperature. The temperature increment is 0.1 K in (a, c, e) and 0.3 K in (b, d, f). The solid lines are fits of the data using Equation (1). (g) Temperature-dependent ${\Lambda }^{ - 1}( T )$. Dots represent experimental data and the curves are fittings of the experimental data to Equation (2). The error bars of the fitting are smaller than the size of the data points. (h) Fitting parameters of Points 1–4. Left axis (blue): zero-temperature $\Lambda _0^{ - 1}$, which is proportional to the superfluid density at $T = 0\ {\mathrm{K}}$; right axis (inner): $\alpha $; right axis (outer): ${T}_{\mathrm{C}}$.

The spatial inhomogeneity of the NSNO film enables us to investigate the correlation between ${T}_{\mathrm{C}}$ and the superfluid density in a statistical way. For a superconducting thin film, the local superfluid density is directly proportional to the diamagnetic susceptibility according to the thin diamagnet film model [51]:


(1) \begin{eqnarray*}
\frac{{\chi ^{\prime}\!\left( z \right)}}{{{\chi }_{\mathrm{s}}}} = - \frac{a}{\Lambda }\left( {1 - \frac{{2\frac{{z - {z}_0}}{a}}}{{\sqrt {1 + 4{{\left( {\frac{{z - {z}_0}}{a}} \right)}}^2} }}} \right),
\end{eqnarray*}

where ${\chi }_{\mathrm{s}}$ represents the SQUID self-susceptibility, *a* represents the effective radius of the field coil, ${z}_0$ represents the offset of the sample–coil distance and $\Lambda $ represents the Pearl length, which is proportional to the inverse of the superfluid density (${\Lambda }^{ - 1} = \frac{d}{2}{\lambda }^{ - 2}$, where *d* represents the film thickness). Figure 3a–f depicts the susceptibility-approach data (the dots) and the fitting curves (the solid lines) of Equation (1) for the selected points. We note that, although the low-temperature-approach data can be well fitted to Equation (1), the fitting curves exhibit non-negligible deviations from the experimental data at some temperatures near ${T}_{\mathrm{C}}$. The relatively poor fitting of the approach data to Equation (1) near ${T}_{\mathrm{C}}$ was also found by [34] and was attributed to the strong paramagnetic response at relatively high temperatures. Besides, the significant spatial inhomogeneity of the superfluid density near ${T}_{\mathrm{C}}$, with a length scale smaller than the Pearl length, could lead to the breakdown of Equation (1) that assumes a homogeneous superfluid density [60].

Figure 3g shows the extracted temperature-dependent superfluid density ${\Lambda }^{ - 1}( T )$ from the susceptibility-approach curves. We observe nearly *T*-linear behavior of ${\Lambda }^{ - 1}$ for $T < 7\ {\mathrm{K}}$ on all points, consistently with the results reported in [36]. The relatively rapid drop of the superfluid density near ${T}_{\mathrm{C}}$ is reminiscent of the Berezinskii–Kosterlitz–Thouless transition in 2D superconductors that originated from the unbinding of the vortex–antivortex pair due to thermal phase fluctuations [61,62]. We further fit the ${\Lambda }^{ - 1}( T )$ relations with a phenomenological formula (see the solid lines in Fig. 3g):


(2) \begin{eqnarray*}
{\Lambda }^{ - 1} = \Lambda _0^{ - 1}{\left( {1 - \frac{T}{{{T}_{\mathrm{C}}}}} \right)}^\alpha ,
\end{eqnarray*}

where $\Lambda _0^{ - 1} = {\Lambda }^{ - 1}( {0\ {\mathrm{K}}} )$ and $\alpha $ represents a parameter that possibly relates to the superconducting gap anisotropy [63,64]. For Points 2 and 3, which are located close to the boundary of the main WDR, their $\alpha $ values are larger than those of Points 1 and 4 (squares in Fig. 3h). Intriguingly, we find that $\Lambda _0^{ - 1}$ (circles) tracks the local ${T}_{\mathrm{C}}$ (diamonds) closely for these points, which suggests a strong correlation between the two quantities.

**Figure 4.**
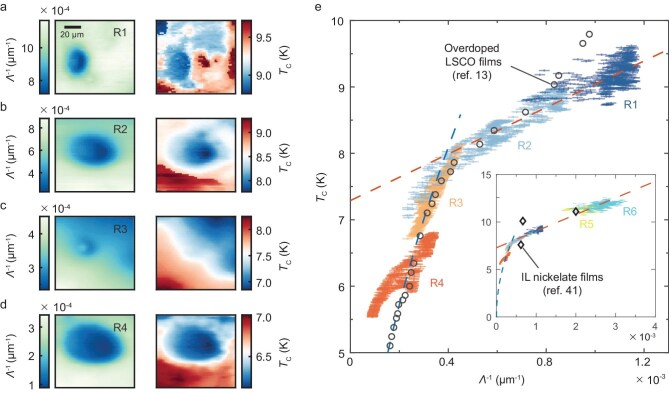
Correlation between superfluid density and local superconducting transition temperature in NSNO. (a–d) Mappings of the inverse of the Pearl length ${\Lambda }^{ - 1}$ at the base temperature ${T}_{{\mathrm{base}}} = 5\ {\mathrm{K}}$ (left column) and the local critical temperature ${T}_{\mathrm{C}}$ (right column) for regions R1–R4. (e) Scaling of ${T}_{\mathrm{C}}$ with ${\Lambda }^{ - 1}$ extracted from (a–d). The colors of the markers represent the scan area from which the data were extracted. The length and the width of markers represent the uncertainties in determining the local ${T}_{\mathrm{C}}$ and ${\Lambda }^{ - 1}$, respectively. Data points with ${T}_{\mathrm{C}}\ > \ 8\ {\mathrm{K}}$ can be fitted with a linear relation ${T}_{\mathrm{C}} = {T}_0 + \alpha {\Lambda }^{ - 1}$, where ${T}_0 = 7.29\ {\mathrm{K}}$, $\alpha = 1.75 \times {10}^3\ {\mathrm{K}}\cdot{\mathrm{\mu m}}$ (the red dashed line), and those with ${T}_{\mathrm{C}}\ < \ 8\ {\mathrm{K}}$ can be fitted with a parabolic relation ${T}_{\mathrm{C}} = \gamma \sqrt {{\Lambda }^{ - 1}} $, where $\gamma = 405\ {\mathrm{K}}\cdot{\mathrm{\mu }}{{\mathrm{m}}}^{1/2}$ (the blue dashed line). For reference, we include the data of overdoped LSCO [13], where the values of ${T}_{\mathrm{C}}$ and ${\Lambda }^{ - 1}$ have been rescaled (circles). The inset provides the ${T}_{\mathrm{C}}$-versus-${\Lambda }^{ - 1}$ data extracted from two scan regions (R5–R6) of the second NSNO film with higher ${T}_{\mathrm{C}}$ (Supplementary Material S8), alongside data from the STO-capped *R*_0.8_Sr_0.2_NiO_2_ films measured by using the mutual inductance technique [41] (diamonds). Here, the ${\Lambda }^{ - 1}$ values for R5–R6 and *R*_0.8_Sr_0.2_NiO_2_ are rescaled by constant factors of 2.9 and 0.15, respectively.

During the scanning process, the nano-SQUID probe moves parallel to the film surface, at a constant scan height of ∼0.8 μm. Hence, the geometry factors in Equation (1), i.e. *a, z* and ${z}_0$, maintain constant values. In this regard, the value of ${\Lambda }^{ - 1}$ for any point in the scan area of Fig. 1c (denoted as R1) with respect to point *i* (*i* = 1–4) is equal to the ratio of their $\chi ^{\prime}$ values. This allows us to transform the$\ \chi ^{\prime}$ image (Fig. 1c) into the ${\Lambda }^{ - 1}$ image, as displayed in the left panel of Fig. 4a (see details in Supplementary Materials S6). Using this procedure, we have obtained the ${\Lambda }^{ - 1}$ images from three other scan areas (denoted as R2–R4), which all exhibit spatial variation at the micron scale (see the left panels in Fig. 4b–d). The corresponding mappings of the local *T*_C_ are provided in the right panels of Fig. 4a–d, showing clear spatial correlation with the ${\Lambda }^{ - 1}$ images.

The ${T}_{\mathrm{C}}$-versus-${\Lambda }^{ - 1}( {{T}_{{\mathrm{base}}}} )$ data extracted from different random areas across the sample exhibit a continuous trend (Fig. 4e). For all the points in these areas, a higher base-temperature superfluid density corresponds to a higher ${T}_{\mathrm{C}}$. In particular, for R1–R2 with ${T}_{\rm C}\ > \ 8\ {\mathrm{K}}$, the data appear to obey a linear relationship with an offset, ${T}_{\rm C} = {T}_0 + \alpha {\Lambda }^{ - 1}$ with ${T}_0\ = \ 7.29\ {\mathrm{K}}$ and $\alpha = 1.75\ \times {10}^3\ {\mathrm{K}}\cdot{\mathrm{\mu m}}$ (the red dashed line), whereas, for R3–R4 with ${T}_{\rm C} < \ 8\ {\mathrm{K}}$, the data suggest a sublinear relationship between ${T}_{\mathrm{C}}$ and ${\Lambda }^{ - 1}$ that can be captured by ${T}_{\mathrm{C}} = \gamma \sqrt {{\Lambda }^{ - 1}} $ with $\gamma = 405\ {\mathrm{K}}\cdot$µm^1/2^ (the blue dashed line). To check whether the ${T}_{\mathrm{C}}$–${\Lambda }^{ - 1}( {{T}_{{\mathrm{base}}}} )$ relationship is robust, we have extracted the ${T}_{\mathrm{C}}$-versus-${\Lambda }^{ - 1}( {{T}_{{\mathrm{base}}}} )$ data from two scan areas (R5–R6) of the second NSNO film with ${T}_{\mathrm{C}}$ ranging from 11.2 to 12.6 K (Supplementary Materials S8). As shown in the inset of Fig. 4e, the data for R5 (yellow markers) and R6 (cyan markers) are in line with the extrapolated linear relationship (the red dashed line) of R1–R2 (see also the enlarged view in Fig. S10e). Moreover, data from the STO-capped *R*_0.8_Sr_0.2_NiO_2_ (*R* = La, Pr and Nd) measured by using the mutual inductance technique [41] also follow a similar trend (diamonds). Here, the ${\Lambda }^{ - 1}$ values for *R*_0.8_Sr_0.2_NiO_2_ and R5–R6 are rescaled by constant factors of 0.15 and 2.9, respectively. The former may be related to the presence of the STO-capping layer in their samples, which has been found to influence the electronic structure of the NSNO [22,65] and thereby potentially affect the superfluid density, whereas, for the two uncapped films studied here, the difference in the ${\Lambda }^{ - 1}$ values is mainly attributed to the difference in their dead-layer thicknesses (${d}_{{\mathrm{dead}}}$) [66]. With the assumption of a negligible ${d}_{{\mathrm{dead}}}$ in the first sample, the rescaling factor of 2.9 indicates ${d}_{{\mathrm{dead}}} \approx 4.8\ {\mathrm{nm}}$ for the second NSNO film, which is close to the reported value of 4.6 nm for some uncapped NSNO films [67].

In order to obtain the relationship between ${T}_{\mathrm{C}}$ and the zero-temperature superfluid density $\Lambda _0^{ - 1} = {\Lambda }^{ - 1}( {T\rightarrow 0\ {\mathrm{K}}} )$, we extrapolate ${\Lambda }^{ - 1}( T )$ at finite temperatures to $T\ = \ 0\ {\mathrm{K}}$ by using both nodeless-gap and nodal-gap models, which have been widely used to analysis the temperature dependence of the superfluid density for high-${T}_{\mathrm{C}}$ superconductors [63,68,69] including IL nickelates [41] (see details in Supplementary Materials S7). Due to the temperature limitations of the scanning SQUID device, it is difficult to determine which model is better for fitting the raw ${\Lambda }^{ - 1}( T )$ data (Fig. S8a). Nevertheless, we find that the ${T}_{\mathrm{C}}( {\Lambda _0^{ - 1}} )$ data obey the similar scaling relationships between ${T}_{\rm C}$ and ${\Lambda }^{ - 1}( {{T}_{{\mathrm{base}}}} )$, regardless of the gap model employed (see Figs S8d and S9c accounting for the errors in extrapolating $\Lambda _0^{ - 1}$). We note that the NSNO film may host multiband or multigap superconductivity, which could result in a kink in ${\Lambda }^{ - 1}( T )$ when the interband coupling is weak [70,71]. However, such a kink is absent in the ${\Lambda }^{ - 1}( T )$ of our samples and those of the NSNO films studied previously [36,41].

Remarkably, the scaling between ${T}_{\rm C}$ and the superfluid density that we observe is conspicuously similar to that observed in overdoped LSCO films when using the bulk mutual inductance technique [13]. By rescaling the values of ${T}_{\mathrm{C}}$ and ${\Lambda }^{ - 1}$ for LSCO (black circles in Fig. 4e), the two datasets overlap almost exactly, except for in the high-${T}_{\rm C}$ region, where the slope of LSCO is larger than that of NSNO. It should be noted that the superfluid density data of LSCO were accumulated from >2000 films prepared over ∼12 years [72]. In contrast, the method of susceptometry imaging combined with spatial statistics, which was proposed for the first time in this work, enables the acquisition of continuous and dense ${T}_{\mathrm{C}}( {{\Lambda }^{ - 1}} )$ data on a single sample containing inhomogeneity. Such a high-efficiency methodology also avoids errors caused by the variations in growth conditions from one sample to another in the bulk measurements [73,74]. A similar method for extracting the ${T}_{\mathrm{C}}$–${\Lambda }^{ - 1}$ relationship from a single sample has been recently applied to iron-based superconductors [16]. On the other hand, we noticed that the variation in the ${T}_{\mathrm{C}}$ of NSNO (from 5.5 to 12.6 K) is less than that of LSCO (from 4 to 45 K) due to the limited maximum ${T}_{\mathrm{C}}$ of NSNO. Future superfluid density measurements on other nickelate compounds with a maximum ${T}_{\mathrm{C}}$ exceeding 20 K, e.g. Sm_1__–_*_x_*_–_*_y_*_–_*_z_*Ca*_x_*Sr*_y_*Eu*_z_*NiO_2_ [75] and Sr- or Pr-doped La_3_Ni_2_O_7_ [76–78], will help to examine whether the scaling relationship extends to ${T}_{\mathrm{C}} > 12.5{\mathrm{\ K}}$.

The linear relationship between ${T}_{\mathrm{C}}$ and the superfluid density has been considered empirical evidence that ${T}_{\mathrm{C}}$ is dominated by the phase stiffness [72,79,80], in contrast to the standard BCS paradigm that ${T}_{\mathrm{C}}$ is dominated by the pairing strength. The long-range phase coherence is destroyed at ${T}_{\mathrm{C}}$ owing to stronger thermal phase fluctuations with decreasing superfluid density. Thus, the observed linear scaling between ${T}_{\mathrm{C}}$ and the superfluid density in R1–R2 may also suggest significant thermal phase fluctuations in NSNO. This is supported by the rapid drop in ${\Lambda }^{ - 1}( T )$ above ${T}_{\mathrm{C}}$ (see Fig. 3g) and also the mutual inductance measurements of IL nickelates [41], and by the crossover from 3 to 1 in the power-law exponent of the current–voltage characteristic curves [81–83]. Furthermore, a recent THz pump-probe study on NSNO also points out substantial superconducting fluctuations near ${T}_{\rm C}$ [84]. For R3 and R4 with ${T}_{\mathrm{C}} < 8\ {\mathrm{K}}$, the sublinear relationship of ${T}_{\mathrm{C}} = \gamma \sqrt {{\Lambda }^{ - 1}} $ can be understood in terms of quantum phase fluctuations within the (3 + 1)D-*XY* universality class with $z\ = \ 1$ as ${T}_{\mathrm{C}}\rightarrow 0\ {\mathrm{K}}$ [11], which has been adopted to understand the similar relationship for overdoped LSCO with ${T}_{\mathrm{C}}\ < \ 12\ {\mathrm{K}}$ [12].

On the other hand, several theoretical studies have shown that the ${T}_{\mathrm{C}}$-versus-superfluid-density relationship in overdoped LSCO can be reproduced in calculations on the *d*-wave BCS theory by considering the effects of disorder scattering [85–87]. However, their calculations either depend critically on the specific band structure of LSCO or involve the anisotropic scattering of apical oxygen vacancies that is absent for the IL nickelates. Whether these disorder scenarios could be extended to explain the quantitative relationship between ${T}_{\mathrm{C}}$ and the superfluid density in NSNO remains unclear. Regardless, the similarity in the ${T}_{\mathrm{C}}$-versus-superfluid-density relationship between cuprate superconductors and IL nickelates suggests a close underlying principle governing the superconductivity of these two high-${T}_{\mathrm{C}}$ families, despite their distinct electronic structures and pairing symmetries.

In summary, we perform local susceptometry on the IL NSNO/STO films and establish a quantitative relation between the local superfluid density and ${T}_{\mathrm{C}}$. We find that their relationship is strikingly similar to that found in overdoped cuprate superconductors. Our findings suggest unconventional pairing beyond the BCS paradigm in IL nickelates and its close ties with cuprates.

## Supplementary Material

nwag068_Supplemental_File
