# Supplementary material for: Correlation between superfluid density and transition temperature in infinite-layer nickelate superconductor Nd1–xSrxNiO2
Source: Natl Sci Rev. 2026 Jan 31;13(8):nwag068. doi: 10.1093/nsr/nwag068 (PMC13116388; doi:10.1093/nsr/nwag068)
Supplement: nwag068_Supplemental_File [file nwag068_supplemental_file.docx]

Supplementary materials for

**Correlation between superfluid density and transition temperature in infinite-layer nickelate superconductor Nd_1-_*_x_*Sr*_x_*NiO_2_**

**Contents**

**S1. Sample preparation and characterization**

**S2. Scanning superconducting quantum interference device microscopy**

**S3. Temperature evolution of the DC magnetic flux image**

**S4. Analyses of the susceptibility images and the pararesistance**

**S5. The ubiquity of the inhomogeneous superconductivity**

**S6. Converting the susceptibility image into the superfluid density image**

**S7. Determination of the zero-temperature superfluid density**

**S8. *T*_c_ versus *Λ*^-1^ scaling of another Nd_1-_*_x_*Sr*_x_*NiO_2_ sample with higher *T*_c_**

**S9. Properties of the bulk NdNiO_2_**

**S1. Sample preparation and characterization**

**Film synthesis.** The perovskite Nd_0.8_Sr_0.2_NiO_3_ epitaxial films were grown on single-crystal SrTiO_3_ (001) substrates using pulsed laser deposition (PLD, Demcon TSST) with a KrF excimer laser (*λ* = 248 nm). SrTiO_3_ (001) substrates with sizes of 5 × 5 mm^2^ (from HeFei KeJing Mater. Ltd.) were etched by HF and annealed at 1050 °C for 2 h to achieve atomic-flat TiO_2_-termination. The TiO_2_-terminated SrTiO_3_ (001) substrates were then pre-annealed in PLD chamber for 1 h at 640 °C with oxygen partial pressure about 5 × 10^-6^ Torr to obtain sharp steps before film growth. In this study, we performed ablation using uniform rectangular laser spots with size of 1.0 × 3.0 mm^2^, which were formed by aperture imaging. The polycrystalline target Nd_0.8_Sr_0.2_NiO_3_ (synthesized by solid-state reaction method from commercial oxide powders of Nd_2_O_3_, SrCO_3_, Ni_2_O_3_ with stoichiometric ratios) were ablated using laser energy density in the range of 1.0–1.3 J cm^-2^ with a frequency of 4 Hz. High-pressure in-situ reflective high-energy electron diffraction (RHEED, Staib Instruments) was used to monitor the film growth process. The growth temperature of the perovskite precursor films was set to 550 ℃~ 650 ℃ and the oxygen partial pressure was kept at 200 mTorr. The ramp rate of the temperature is strictly controlled at 10 ℃ min^-1^. All samples mentioned in the text were grown without SrTiO_3_ capping layer.

**Topotactic reduction.** The as-grown film is loosely wrapped in aluminum foil to avoid direct contact with the reducing agent, placed in a Pyrex glass tube containing 0.1 g CaH_2_ powder and vacuum sealed (pressure < 0.1 mTorr). The topotactic reduction was performed at the optimized conditions based on our previous studies, i.e., at the temperature of 300 °C and reduction time period of 2 h with the ramp rate of the temperature fixed at 10 ℃ min^-1^. For air stability test, the as-grown superconducting film was stored in vacuum condition right after the structural and electrical measurements.


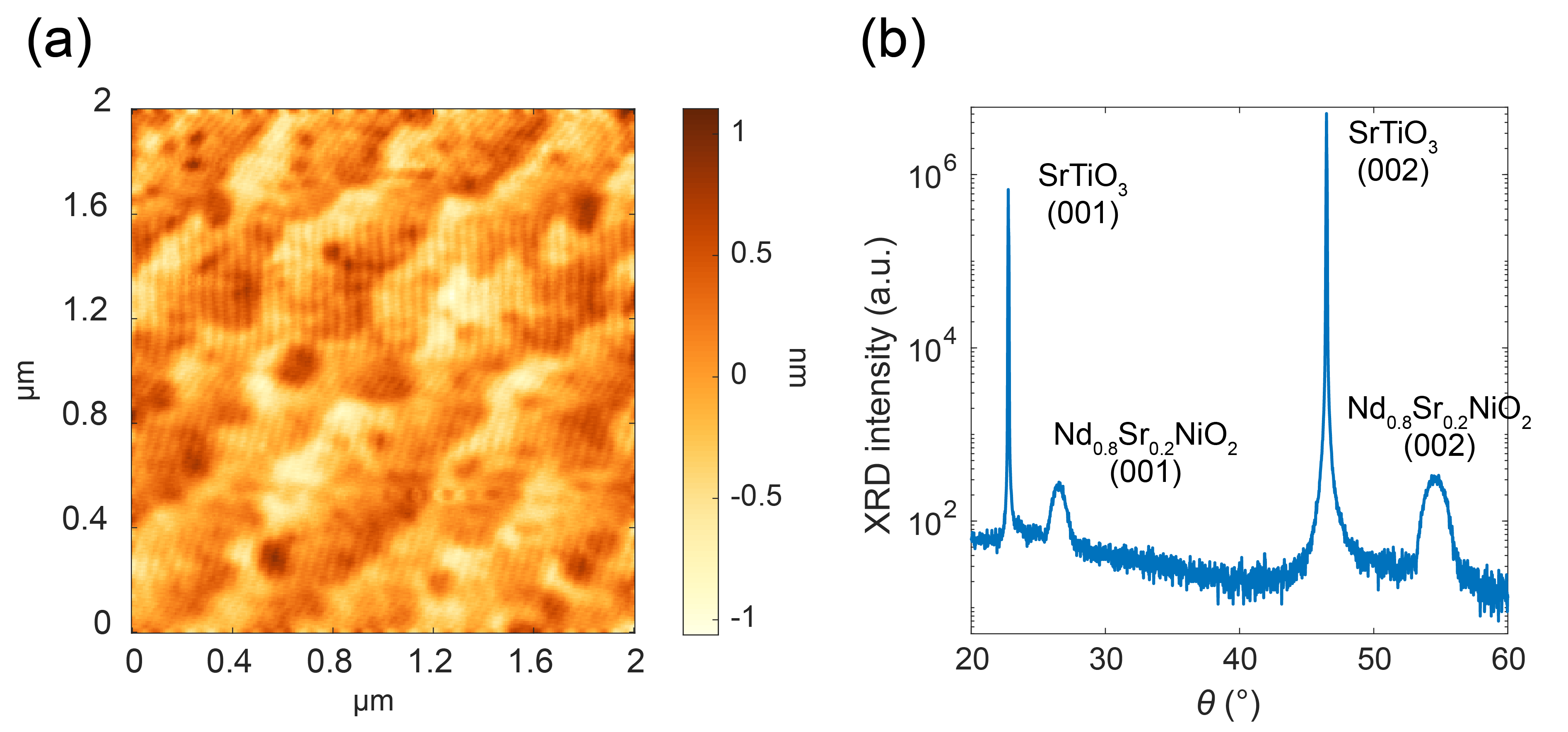


**FIG. S1 Structural characterization of the Nd_1-_*_x_*Sr*_x_*NiO_2_ film.** (a) Atomic force microscopy (AFM) image of the film. Clear terraces indicate the high quality of the film. (b) X-ray diffraction of the sample. Strong intensity peaks emerge at *θ* = 26.5° and 54.3°, corresponding to (001) and (002) peak of the lattice, respectively. The full width at half maximum (FWHM) of the rocking curve is 0.08°, indicating high crystalline quality.


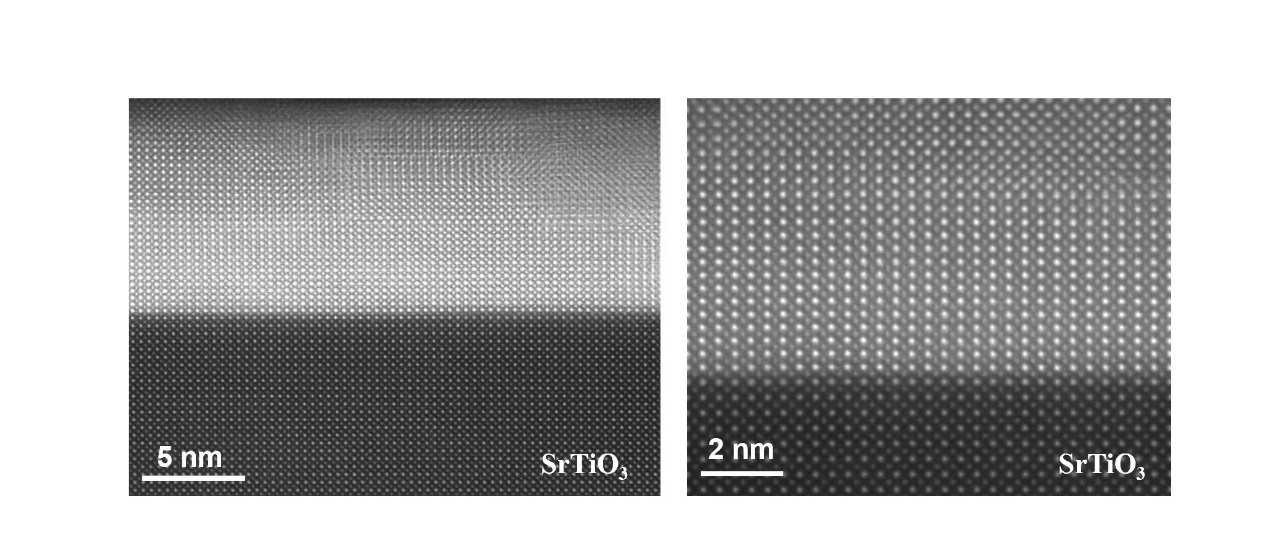


**Fig S2. STEM cross-sectional images of the sample.** The darker and brighter regions correspond to the SrTiO_3_ substrate and the Nd_1-_*_x_*Sr*_x_*NiO_2_ (NSNO) film, respectively. In spite of some Ruddlesden-Popper (RP) defects as denoted by the red frames, the lattice of the film shows good uniformity and matches that of the substrate. These RP defects may be the potential origin of the observed WDR.


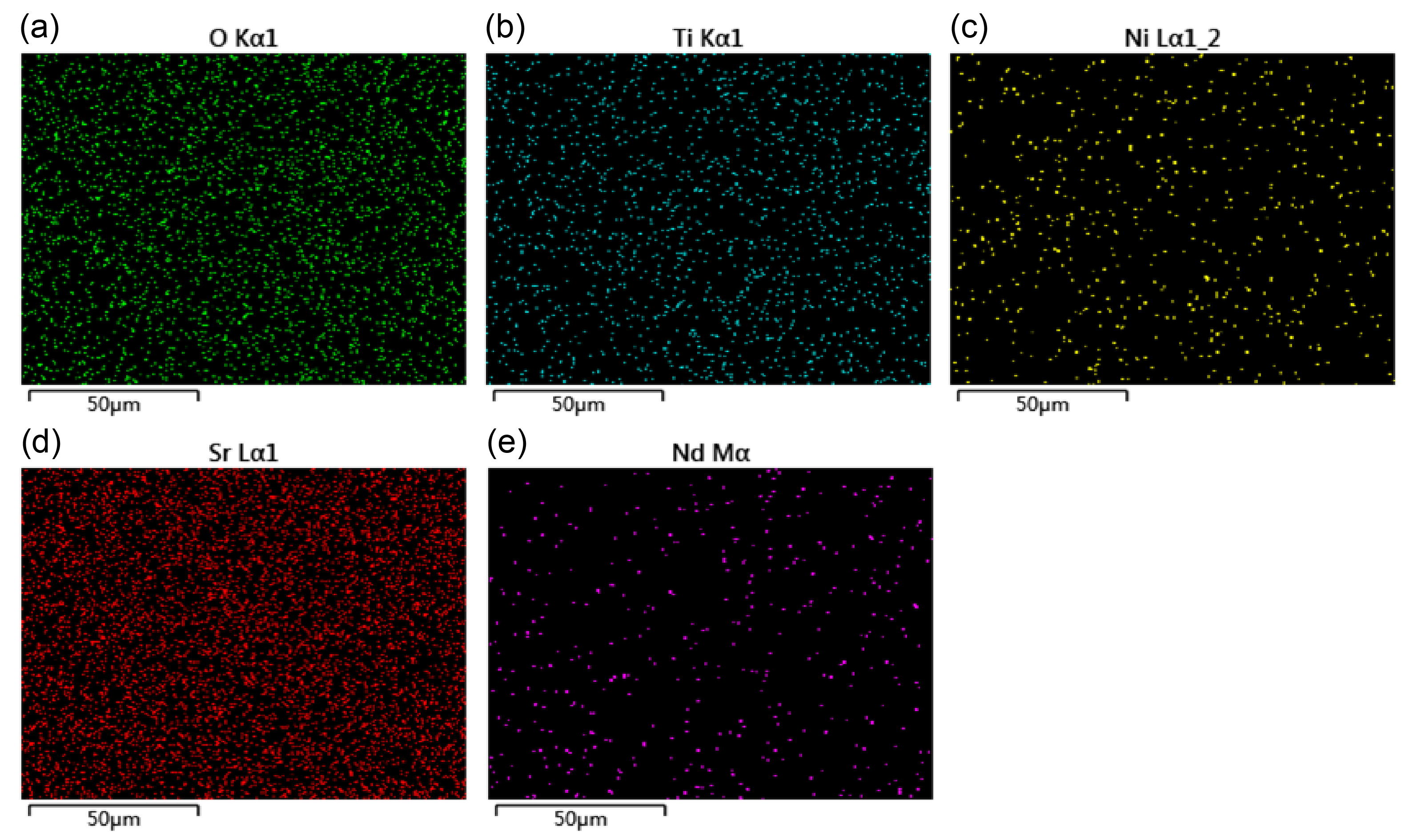


**FIG. S3 Typical X-ray diffraction energy dispersive spectra (XRD-EDS) mappings of the NSNO film.**

**S2. Scanning superconducting quantum interference device microscopy**

To characterize the spatial distribution of the superconductivity of the NSNO film, we employ scanning superconducting quantum interference device (sSQUID) magnetometry and susceptometry [1–9], which has a high magnetic flux sensitivity without applying an external magnetic field. Our nano-SQUID device consists of a two-junction SQUID involving two pickup coils with diameters of 2 μm, arranged in a gradiometric design to cancel out any uniform external field including the geomagnetic field. Magnetic flux (*Φ*) across the pickup loop is linearly converted into voltage signals through flux-locked loop [10]. By applying an excitation current to the field coil, the real part of the AC susceptibility (*χ'*) can be obtained through demodulation of the in-phase component of the feedback of the pickup coil [11]. The susceptibility approach curves *χ'*(*z*), obtained by scanning the distance (*z*) between the nano-SQUID and the sample, allow us to extract superfluid density from a superconducting thin film. To be able to conduct measurements above the *T*_c_ of NSNO, the nano-SQUID device is thermally isolated from the temperature-variable sample stage to maintain a working temperature of *T* = 4.60 K. A μ-metal is employed to further shield the geomagnetic field and a homemade coil surrounding the cryostat enables fine tuning of the external field.

**S3. Temperature evolution of the DC magnetic flux image**

Fig. S4 presents the DC magnetic flux of the same region of Fig. 2. The puddle-like patterns persist above the bulk *T*_c_, suggesting that they do not originate from the superconductivity.


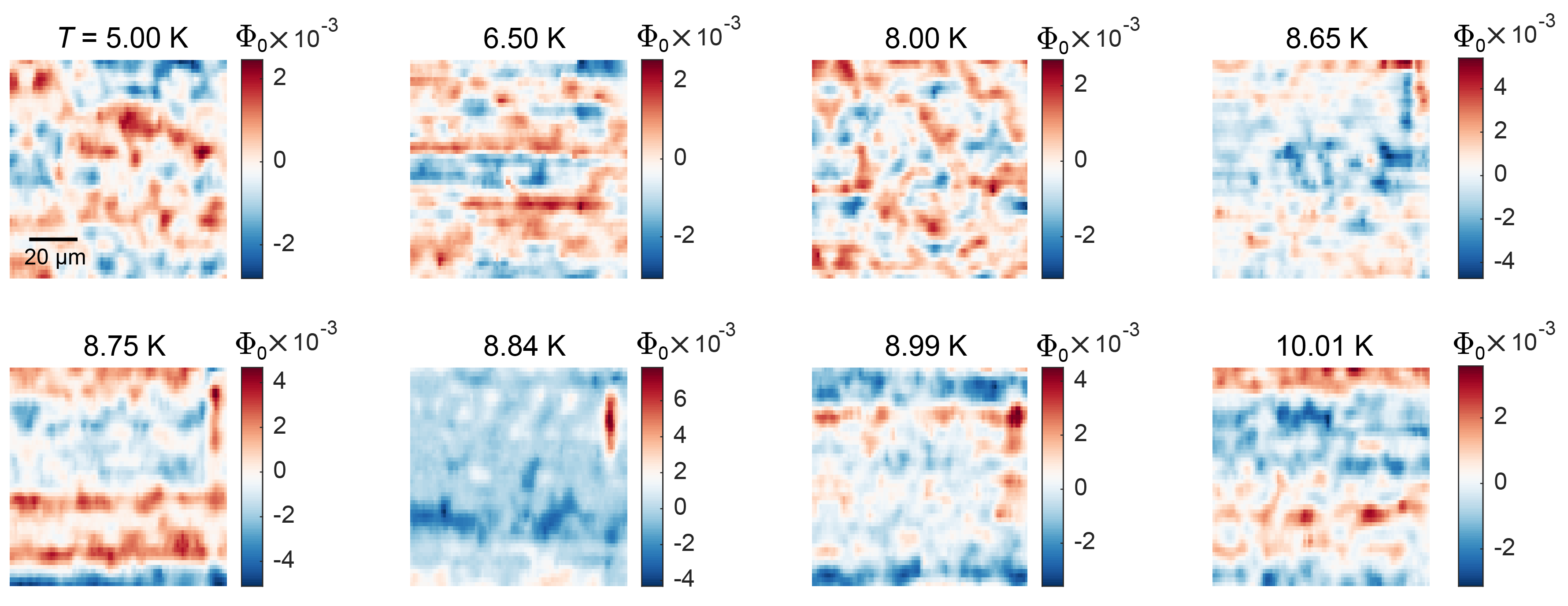


**FIG. S4 DC magnetic flux images of the same region of [FIG. 1(c)] at different temperatures.** Due to flux jumps during the scanning, some lines show conspicuous offsets.

**S4. Analysis of the susceptibility images and the pararesistance**

To evaluate the typical size of the superconducting patches with constant diamagnetic susceptibilities, *l*_p_, we calculate the autocorrelation maps at different temperatures from Fig. 2 using the following formula [12]:

$\gamma(u,v)=\frac{\sum_{x,y} [f(x,y)-\bar{f}_{u,v}][t(x-u,y-v)-\bar{t}]}{\sqrt{\sum_{x,y} {[f(x,y)-\bar{f}_{u,v}]}^{2}\sum_{x,y} {[t(x-u,y-v)-\bar{t}]}^{2}}}$, (S1)

where $\gamma(u,v)$ is the autocorrelation, $f(x,y)$ the original susceptometry image, $\bar{t}$ the mean value of the template (still the susceptometry image), $\bar{f}_{u,v}$ the mean value of $f(x,y)$ in the region of the template. The results are shown in Fig. S5. The *l*_p_ is determined as the full-width-at-half-maximum of the central peak of the radial profile [$\int_{0}^{2\pi} \gamma(\rho,\varphi)d\varphi$ where *ρ* is the radial distance and *φ* is the polar angle] of the autocorrelation maps. The dependence of *l*_p_ on temperature are shown by the blue circles in Fig. S5(a). It is found that *l*_p_ decreases from a temperature independent value (~ 13 μm) to a small but finite value (~ 1 μm) as temperature is increased across the bulk *T*_c_ of 8.6 K, in sharp contrast to the disappearance of *l*_p_ at bulk *T*_c_ for a homogeneous superconductor.

We conduct an Aslamasov-Larkin fluctuation analysis on the pararesistance data of the IL nickelate superconductor sample, and the results are shown in Fig. S6(a). The pararesistance is defined as $\Delta R_{\text{s}}(T)=\frac{1}{\Delta G_{\text{s}}(T)}=\frac{1}{\frac{1}{R_{\text{s}0}(T)}-\frac{1}{R_{\text{fit}}(T)}}$, where *R­*_s0_ (*T*) is the experimental resistance data, and *R­*_fit_ (*T*) is the fitting of the normal-state resistance with linear-in-temperature behavior, i.e., *R­*_fit_ (*T*) = *AT* + *B*, with the fitted parameters *A* = 1.851, *B* = 124.5 [see the blue dashed line in Fig. 1(a)]. Above bulk *T*_c_, the temperature dependence of pararesistance can be well fitted with the zero-dimensional Aslamasov-Larkin formula, i.e., $\Delta G_{s}{}^{-1}\propto\left( T-T_{c,0D} \right)^{2}$ (the brown line), where *T*_c, 0D_ is the fluctuational critical temperature, indicating the existence of small superconducting patches at relatively high temperatures [Fig. S6(b)].


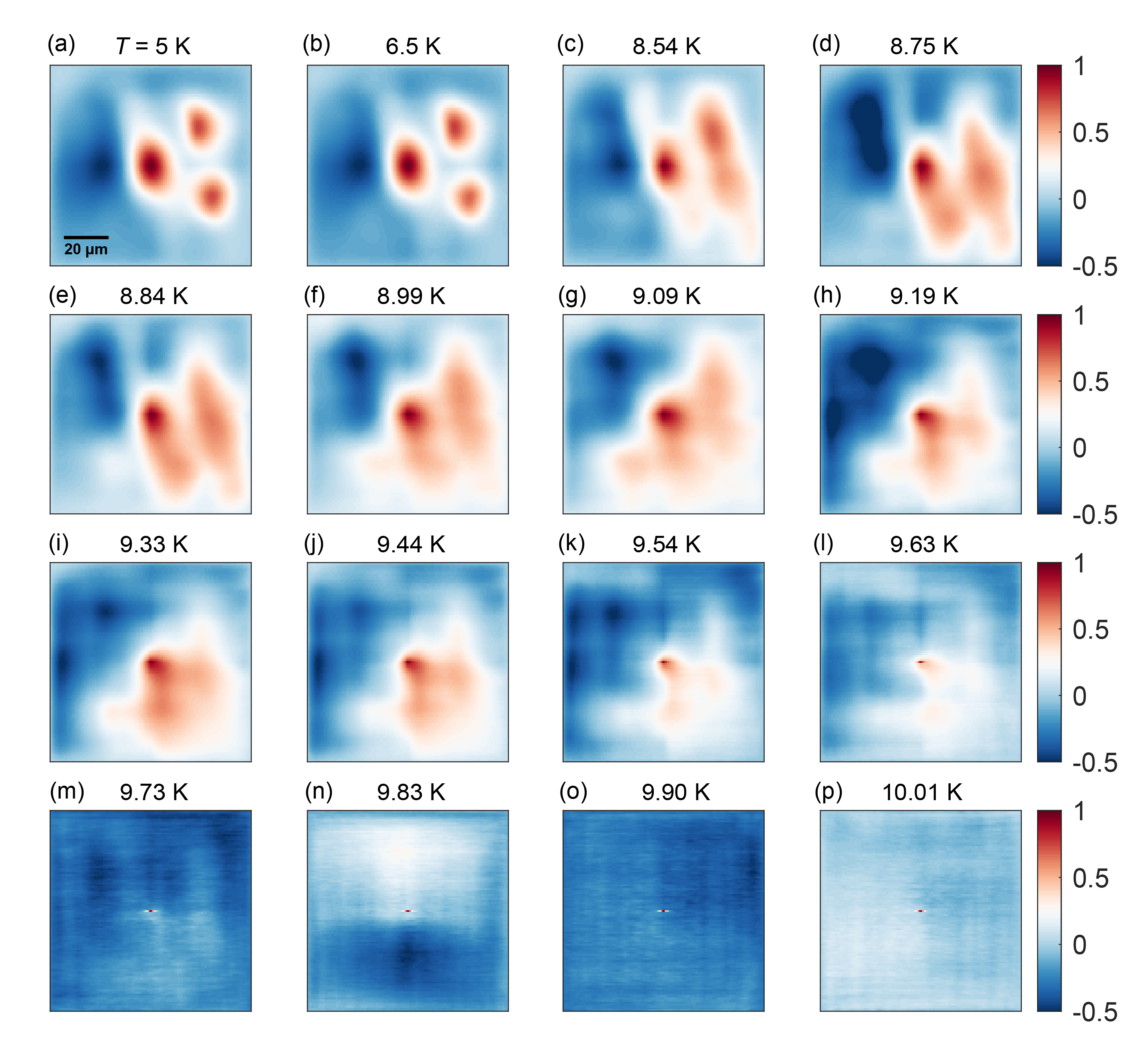


**FIG. S5 Temperature evolution of the autocorrelation plots of the susceptometry images.** The autocorrelation is calculated from Fig. 2 in the main text. All panels share the same color scale.


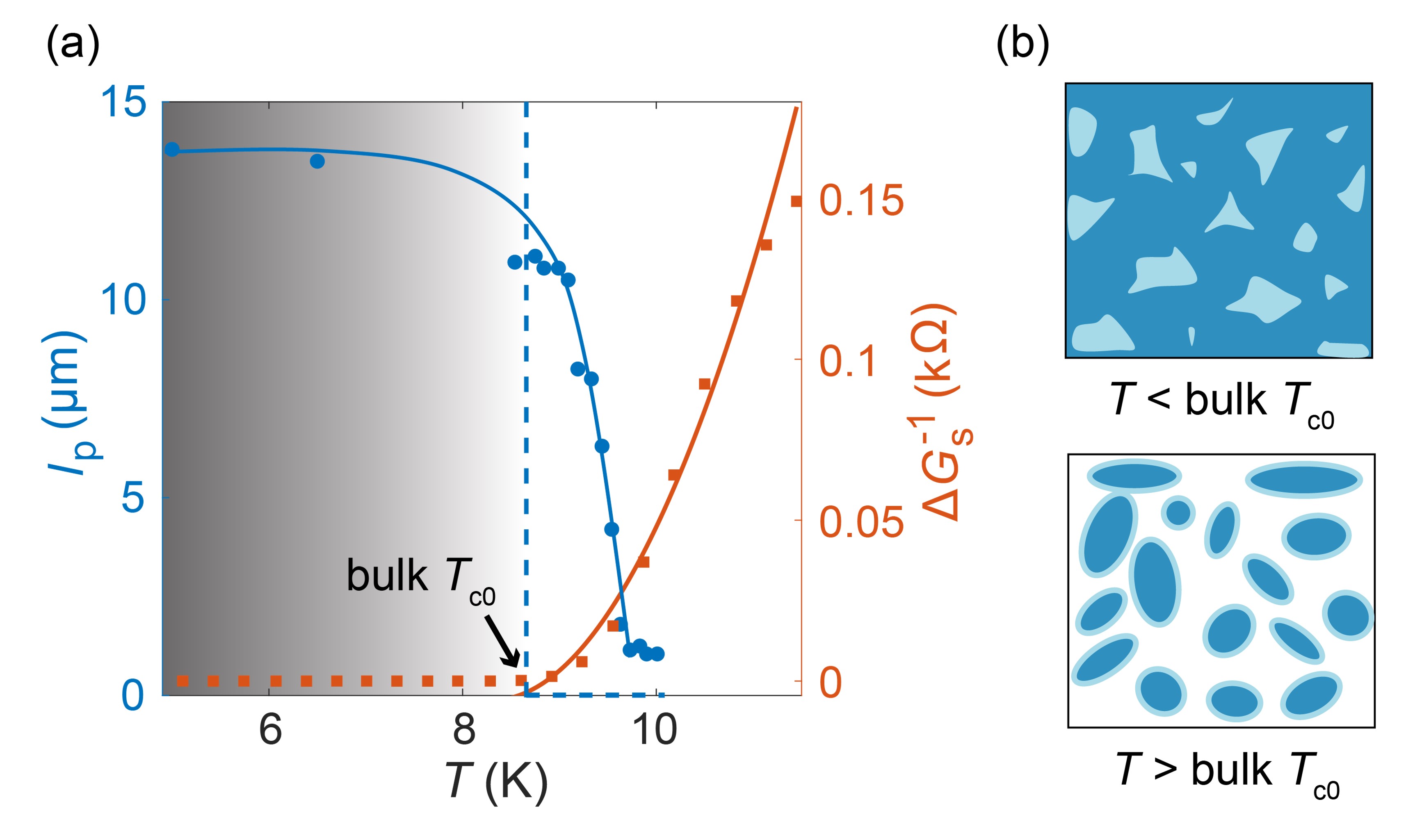


**FIG. S6 Temperature dependence of the inhomogeneous superconductivity.** (a) Left axis (blue): temperature evolution of the typical size of the superconducting patches, *l*_p_, obtained from the autocorrelation maps of Fig. S5. The blue solid line is a guide to the eye. Blue dashed line: temperature dependence of *l*_p_ of an ideal *homogeneous* superconductor. It is comparable to the lateral size of the film (~mm) below *T*_c_, and drops to zero at *T*_c._ Right axis (brown): pararesistance of the NSNO film. The brown solid line shows the fitting of data with zero-dimensional Aslamasov-Larkin formula [$\Delta G_{s}{}^{-1}\propto\left( T-T_{c,0D} \right)^{2}$], where *T*_c, 0D_ is the fluctuational critical temperature. The gray shaded region indicates the region where *T* < bulk *T*_c_. (b) Schematic illustration of the inhomogeneous superfluid density in NSNO film for *T* < bulk *T*_c_ (upper panel) and *T* > bulk *T*_c_ (lower panel).

**S5. Susceptometry imaging of other areas of the sample**

Fig. S7 shows the susceptibility image of different areas of the film measured at *T* = 5 K, all of which exhibit significant inhomogeneity.


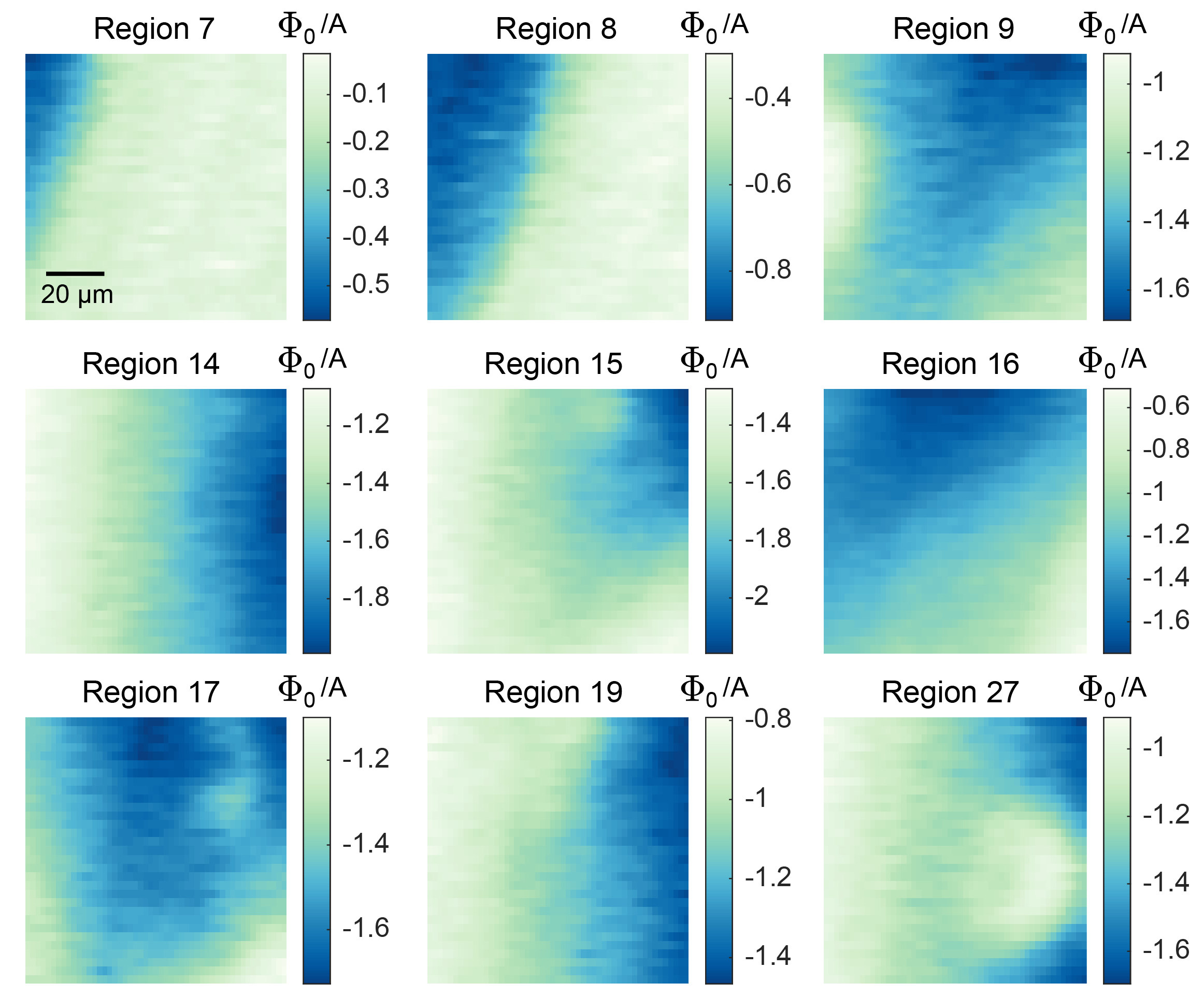


**FIG. S7 Susceptibility mapping of different regions of the film.**

**S6. Converting the susceptibility image into the superfluid density image**

According to Eq. (1) in the main text, $\Lambda^{-1}$ is directly proportional to $\chi'$ for a fixed scan height $z_{\mathrm{scan}}$ [13]:

$$\begin{aligned} \Lambda^{-1}=-C\chi^{'}.\#\left( S2 \right) \end{aligned}$$

Here, $C$ is a temperature-independent constant given by

$$\begin{aligned} C=\left[ \chi_{s}a\left( 1-\frac{2\frac{z_{\mathrm{scan}}-z_{0}}{a}}{\sqrt{1+4\left( \frac{{z_{\mathrm{scan}}-z}_{0}}{a} \right)^{2}}} \right) \right]^{-1},\#\left( S3 \right) \end{aligned}$$

where $\chi_{s}\approx415 \Phi_{0}/A$ is the SQUID self-susceptibility, $a$ is the effective radius of the field coil, and $z_{0}$ is the offset of sample-coil distance. For each scan area shown in the main text, we have measured the susceptibility approach curve $\chi_{\mathrm{ref}}^{'}(z,T)$ at a reference point [see e.g., Fig. 3(b) in the main text]. By fitting the $\chi_{\mathrm{ref}}^{'}(z,T)$ data to Eq. (1), $\Lambda_{\mathrm{ref}}^{-1}(T)$ and the geometric parameters of $a$ and $z_{0}$ can be extracted simultaneously. The typical values of $a$ and $z_{0}$ are 5.4 $\mu m$ and 0.3 $\mu m$, respectively, corresponding to $C\approx5.5\times{10}^{-4} \Phi_{0}/(A\cdot\mu m)$. Assuming that $C$ does not vary significantly during the scanning process, we can evaluate $\Lambda^{-1}(T)$ at other points in the scan area using the relation

$$\begin{aligned} \Lambda^{-1}\left( T \right)=\Lambda_{\mathrm{ref}}^{-1}\left( T \right)\frac{\chi^{'}\left( z_{\mathrm{scan}},T \right)}{\chi_{\mathrm{ref}}^{'}\left( z_{\mathrm{scan}},T \right)}.\#\left( S4 \right) \end{aligned}$$

This relation allows us to convert the $\chi'$ image into the $\Lambda^{-1}$ image at a finite temperature *T* [see e.g., the left panels in Figs. 4(a-d)].

**S7. Determination of the zero-temperature superfluid density**

To obtain $\Lambda_{0}^{-1}$ across the scan area, we first extrapolate the $\Lambda_{\mathrm{ref}}^{-1}\left( T \right)$ data (equivalent to the $\chi_{\mathrm{ref}}^{'} \left( z_{\mathrm{scan}},T \right)$ data with known *C*) at the reference point to $T=0 K$ ($\Lambda_{ref,0}^{-1}$) using either the nodeless-gap model [14,15] [the blue solid line in Fig. S8(a)]:

$\chi'(T)=\chi'(0)\left( 1-\sqrt{\frac{\pi\Delta_{\min}}{2k_{B}T}}e^{-\frac{\Delta_{\min}}{k_{B}T}} \right)$ (S5a)

or the nodal-gap model [16–18] [the red dashed line in Fig. S8(a)]:

$\chi^{'}\left( T \right)=\chi^{'}\left( 0 \right)\left( 1-c_{2}\frac{T^{2}}{T+T^{**}} \right)$. (S5b)

Here *Δ*_min_ is the minimum superconducting gap, *c*_2_ is the fitting parameter, and *T*^**^ is a characteristic temperature. The same gap model is then used to extrapolate $\chi^{'}\left( z_{\mathrm{scan}},T \right)$ in the scan area to $T=0 K$ [$\chi^{'}\left( z_{\mathrm{scan}},0 \right)$], as displayed in Fig. S8(a). Finally, $\Lambda_{0}^{-1}$ in the scan area can be calculated based on the ratio of $\chi^{'}\left( z_{\mathrm{scan}},0 \right)$ to $\chi_{\mathrm{ref}}^{'}(z_{\mathrm{scan}},0)$:

$$\begin{aligned} \Lambda_{0}^{-1}=\Lambda_{ref,0}^{-1}\frac{\chi^{'}\left( z_{\mathrm{scan}},0 \right)}{\chi_{\mathrm{ref}}^{'}(z_{\mathrm{scan}},0)}.\#\left( S6 \right) \end{aligned}$$

The uncertainty in $\Lambda_{0}^{-1}$ is taken as the difference between the values obtained using the nodeless-gap and the nodal-gap fits.

We note that the recent mutual inductance measurements on NSNO/STO [19] show that the superfluid density saturates below 0.2 *T*_c_. This finding is in line with the previous superfluid density measurements on NSNO/STO using sSQUID [20], THz conductivity [21], and tunnel diode oscillator [22] techniques. Collectively, these observations indicate that the minimum gap size $\Delta_{\min}$ should be less than approximately $2k_{B}T_{c}$ for the nodeless-gap fit (Eq. S5a). Therefore, we set the upper bound $\Delta_{\min}\leq2k_{B}T_{c}$ when extrapolating $\Lambda_{0}^{-1}$ using the nodeless-gap fit.

We find that for both fits, the obtained 𝑇_c_(*Λ*_0_^-1^) data [Figs. S8(b-c)] can be captured by a linear relation for *T*_c_ > 8 K [the red dashed lines] and a square root relation for *T*_c_ < 8 K [the blue dashed lines], consistent with Fig. 4(e) in the main text. Fig. S8(d) provides the average of two fitting results, with the error bars of *Λ*_0_^-1^ (the width of the markers) determined by the difference between the values obtained using the two fits.

Besides, we note that the susceptibility of our samples exhibits *T*-linear behavior down to the base temperature of 5 K. Hence, if the nodal-gap model applies to NSNO, the quadratic-to-linear crossover temperature *T*^**^ must lie below 5 K, and may not function effectively as a fitting parameter in the present data window. To evaluate the uncertainty associated with the nodal extrapolation, we calculate the *Λ*_0_^-1^ by fitting the *χ'*(*T*) data using Eq. S5b with constraints of *T*^**^<*T*_c_ [Fig. S9(a)] and *T*^**^ = 0 K [Fig. S9(b)], respectively. For both cases, the obtained *T*_c_ versus *Λ*_0_^-1^ exhibits similar scaling relations with those shown in Fig. 4(e). This is more evident from Fig. S9(c) that depicts the average of the two cases, where the error bars for *Λ*_0_^-1^ (the width of markers) are determined by the differences between the values of them.


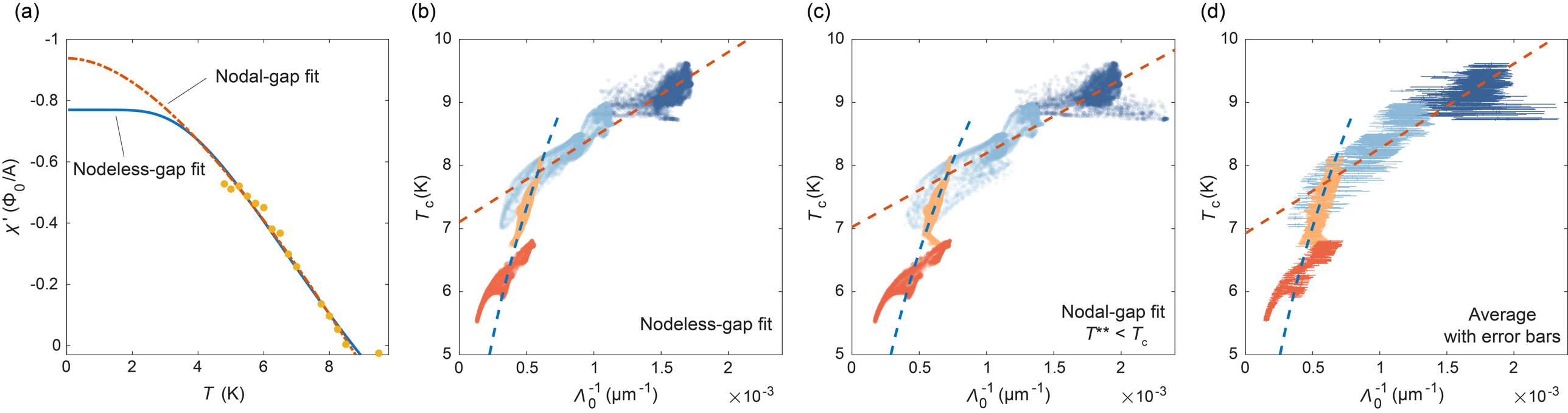


**FIG. S8 Dependence of *T*_c_ on zero-temperature superfluid density in NSNO.** (a) Representative *χ'*(*T*) data of a point in the scanned region. The solid and dashed lines represent the best fits using Eq. S5a and Eq. S5b to the *χ'*(*T*) data, respectively. (b-d) Extrapolated *T*_c_-*Λ*^-1^(0) relation for R1-R4 obtained using the nodeless-gap fit, the nodal-gap fit assuming *T*^**^ < *T*_c_, and the average of the two fitting results, respectively. For panel (d), the length and the width of markers represent the uncertainties in determining local *T*_c_ and *Λ*_0_^-1^, respectively. The latter is determined by the difference between the *Λ*_0_^-1^ values of (b) and (c). The red and blue dashed lines represent the linear fit and the parabolic fit of the data, respectively.


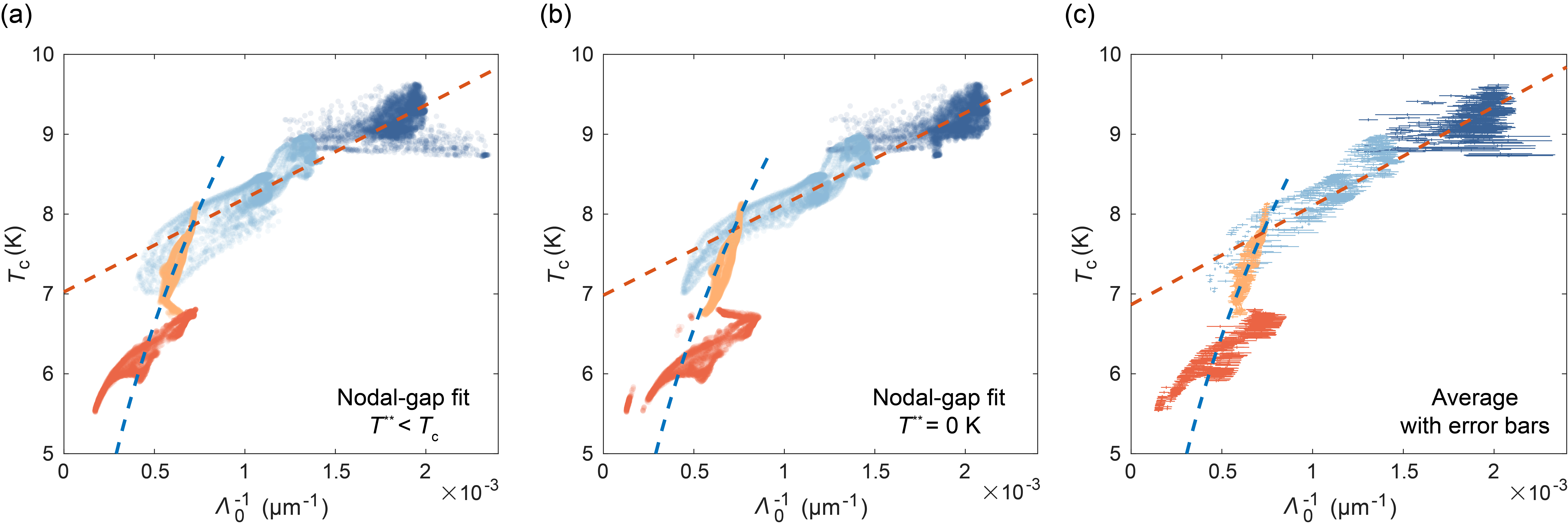


**FIG. S9 *T*_c_ versus zero-temperature superfluid density of NSNO obtained using the nodal-gap fit.** (a-b) Extrapolated *T*_c_-*Λ*^-1^(0) relation for R1-R4 obtained using the nodal-gap fit with constraints of *T*** < *T*_c_, *T*** = 0 K, respectively. (c) is the average of (a) and (b), where the length and the width of markers represent the uncertainties in determining local *T*_c_ and *Λ*_0_^-1^, respectively. The latter is determined by the difference between the *Λ*_0_^-1^ values of (a) and (b). The red and blue dashed lines represent the linear fit and the parabolic fit of the data, respectively.

**S8. *T*_c_ versus *Λ*^-1^ scaling of another Nd_1-_*_x_*Sr*_x_*NiO_2_ sample with higher *T*_c_**

We have performed the sSQUID characterization on another NSNO film with a higher *T*_c_ than the sample presented in the main text. By performing spatial statistics for two scanned regions [Figs. S10 (a-d)], we find the *T*_c_ versus *Λ*^-1^ data can also be fitted with a linear relation [Fig. S10(e)]. Moreover, by rescaling the *Λ*^-1^ values by a factor of 2.9 to account for the difference in the thickness of the dead layer between the two NSNO films [23], we find that the data points align well with the extrapolated linear relation (the red dashed line in Fig. S10(e)) of the first NSNO film [see also the inset of Fig. 4(e)].


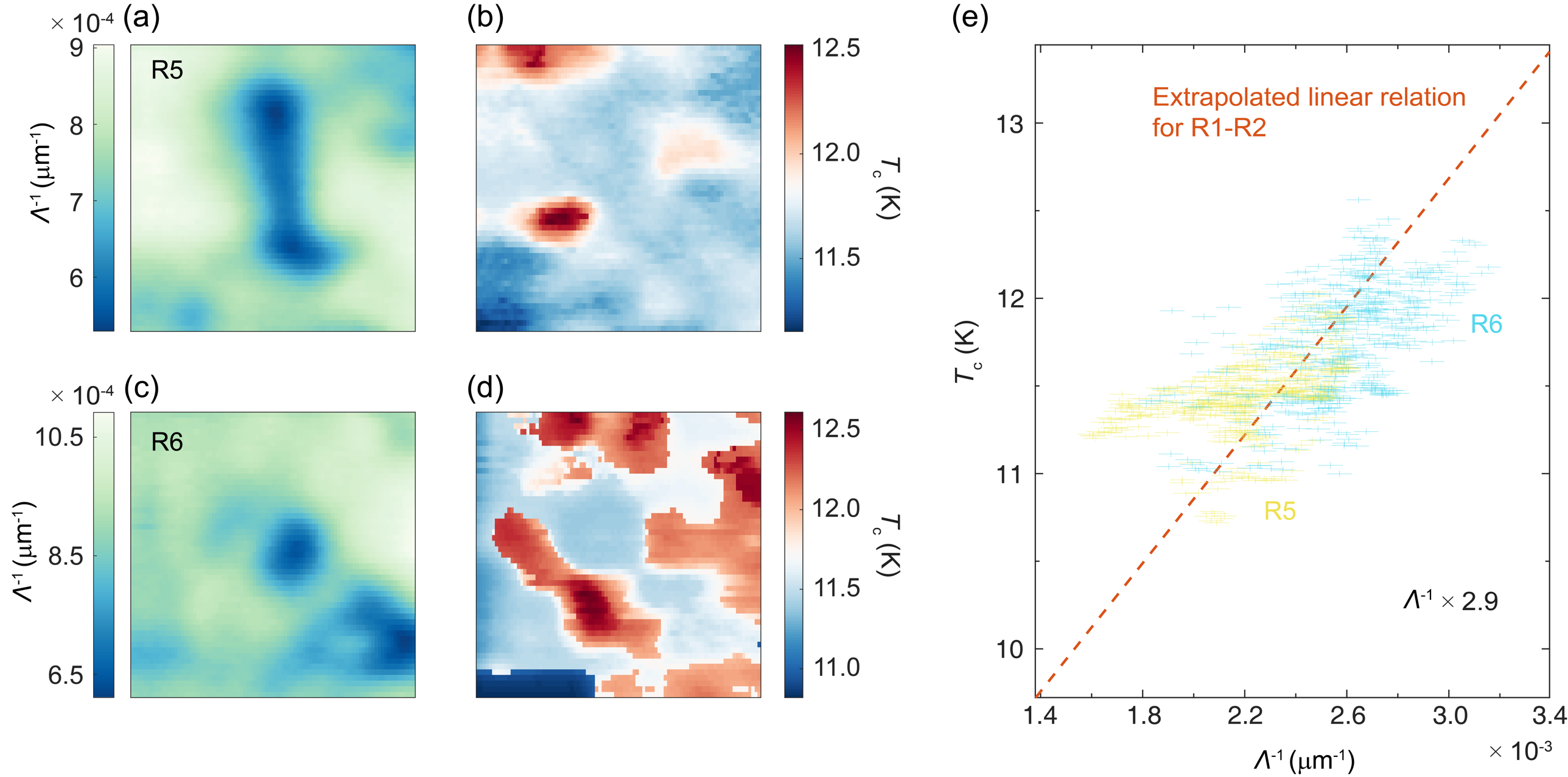


**FIG. S10 Susceptometry and scaling of another NSNO film.** (a-b) Mappings of *Λ*^-1^ and *T*_c_ of a 90 × 90 μm^2^ region for another NSNO film with higher *T*_c_, respectively. The scanned region is denoted as R5. (c-d) Mappings of *Λ*^-1^ and *T*_c_ of another 90 × 90 μm^2^ region, respectively. The scanned region is denoted as R6. (e) *T*_c_(*Λ*^-1^) relation of the two scanned regions extracted from (a-d). Here the *Λ*^-1^ values are rescaled by a factor of 2.9 to account for the difference in the thickness of the dead layer between this film and the first NSNO film. The data points are in line with the extrapolated linear relation (the red dashed line) for the first NSNO film.

**S9. Properties of the bulk NdNiO_2_**

We also test NdNiO_2_ film without strontium doping. The susceptibility shows no significant change with decreasing bender distance, indicating the absence of superconducting diamagnetism. External fields bring offsets in susceptibility baselines, but do not affect the trends.


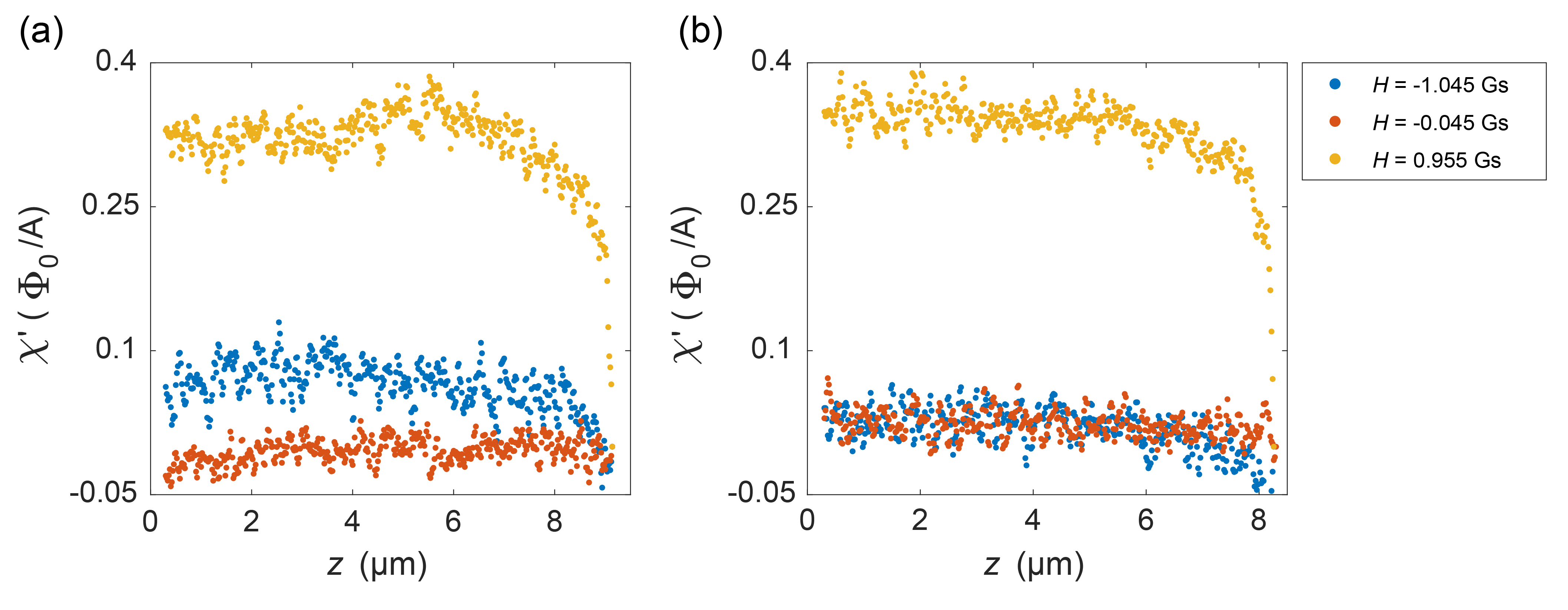


**FIG. S11 Susceptibility approach data of 10 nm-thick NdNiO_2_ films under different external fields.** (a) Approach data of the film without SrTiO_3_ capping. (b) Approach data of the film with 5 nm-thick SrTiO_3_ capping.

**References**

1. Hilgenkamp H, Ariando, Smilde H-JH *et al.* Ordering and manipulation of the magnetic moments in large-scale superconducting *π*-loop arrays. *Nature* 2003; **422**: 50–3.

2. Kirtley JR, Tsuei CC, Ariando *et al.* Angle-resolved phase-sensitive determination of the in-plane gap symmetry in YBa_2_Cu_3_O_7−_*_δ_*. *Nat Phys* 2006; **2**: 190–4.

3. Frolov SM, Stoutimore MJA, Crane TA *et al.* Imaging spontaneous currents in superconducting arrays of *π*-junctions. *Nat Phys* 2008; **4**: 32–6.

4. Finkler A, Vasyukov D, Segev Y *et al.* Scanning superconducting quantum interference device on a tip for magnetic imaging of nanoscale phenomena. *Rev Sci Instrum* 2012; **83**: 073702.

5. Vasyukov D, Anahory Y, Embon L *et al.* A scanning superconducting quantum interference device with single electron spin sensitivity. *Nat Nanotechnol* 2013; **8**: 639–44.

6. Pan YP, Wang SY, Liu XY *et al.* 3D nano-bridge-based SQUID susceptometers for scanning magnetic imaging of quantum materials. *Nanotechnology* 2019; **30**: 305303.

7. Zhang IP, Palmstrom JC, Noad H *et al.* Imaging anisotropic vortex dynamics in FeSe. *Phys Rev B* 2019; **100**: 024514.

8. Noad H, Spanton EM, Nowack KC *et al.* Variation in superconducting transition temperature due to tetragonal domains in two-dimensionally doped SrTiO_3_. *Phys Rev B* 2016; **94**: 174516.

9. Bert JA, Nowack KC, Kalisky B *et al.* Gate-tuned superfluid density at the superconducting LaAlO_3_/SrTiO_3_ interface. *Phys Rev B* 2012; **86**: 060503.

10. Pan YP, Zhu JJ, Feng Y *et al.* Improving spatial resolution of scanning SQUID microscopy with an on-chip design. *Supercond Sci Technol* 2021; **34**: 115011.

11. Clem JR, Coffey MW. Vortex dynamics in a type-II superconducting film and complex linear-response functions. *Phys Rev B* 1992; **46**: 14662–74.

12. Lewis JP. Fast Template Matching. *Proceedings of Vision Interface ’95 Conference*. Quebec City, Canada: Canadian Image Processing and Pattern Recognition Society, 1995, 120–3.

13. Kirtley JR, Kalisky B, Bert JA *et al.* Scanning SQUID susceptometry of a paramagnetic superconductor. *Phys Rev B* 2012; **85**: 224518.

14. Hashimoto K, Shibauchi T, Kato T *et al.* Microwave Penetration Depth and Quasiparticle Conductivity of PrFeAsO 1 − y Single Crystals: Evidence for a Full-Gap Superconductor. *Phys Rev Lett* 2009; **102**: 017002.

15. Prozorov R, Giannetta RW. Magnetic penetration depth in unconventional superconductors. *Supercond Sci Technol* 2006; **19**: R41–67.

16. Božović I, He X, Wu J *et al.* Dependence of the critical temperature in overdoped copper oxides on superfluid density. *Nature* 2016; **536**: 309–11.

17. Skinta JA, Kim M-S, Lemberger TR *et al.* Evidence for a Transition in the Pairing Symmetry of the Electron-Doped Cuprates La_2-_*_x_*Ce*_x_*CuO_4-_*_y_* and Pr_2-_*_x_*Ce*_x_*CuO_4-_*_y_*. *Phys Rev Lett* 2002; **88**: 207005.

18. Hirschfeld PJ, Goldenfeld N. Effect of strong scattering on the low-temperature penetration depth of a *d*-wave superconductor. *Phys Rev B* 1993; **48**: 4219–22.

19. Harvey SP, Wang BY, Fowlie J *et al.* Evidence for nodal superconductivity in infinite-layer nickelates. *Proc Natl Acad Sci USA* 2025; **122**: e2427243122.

20. Shi RA, Wang BY, Iguchi Y *et al.* Scanning SQUID study of ferromagnetism and superconductivity in infinite-layer nickelates. *Phys Rev Mater* 2024; **8**: 024802.

21. Cheng B, Cheng D, Lee K *et al.* Evidence for *d*-wave superconductivity of infinite-layer nickelates from low-energy electrodynamics. *Nat Mater* 2024; **23**: 775–81.

22. Chow LE, Sudheesh SK, Luo ZY *et al.* Pairing symmetry in infinite-layer nickelate superconductor. 2022, DOI: 10.48550/arxiv.2201.10038.

23. Zhang R, Zhao Z, Qin M *et al.* Determining the Thickness of the Dead Layer in Superconducting Film Using a Two-Coil Mutual-Inductance Technique. *Phys Rev Appl* 2022; **17**: 054034.
